# Suspected brain metastasis from lung cancer mimicking intracerebral hemorrhage: A case report

**DOI:** 10.1097/MD.0000000000010106

**Published:** 2018-03-09

**Authors:** Rui Ma, Shao-Kuan Fang, Shuai Hou, Xue Wang, Hong-Mei Meng

**Affiliations:** Department of Neurology and Neuroscience Center, First Hospital of Jilin University, Jilin, P.R. China.

**Keywords:** brain metastasis, intracerebral hemorrhage, lung cancer

## Abstract

**Rationale:**

Hemorrhage rarely occurs in a solitary brain metastasis from lung carcinoma.

**Patient concerns:**

We report on a 54-year-old man who presented with a severe headache for 4 days.

**Diagnoses:**

Based on computed tomography and magnetic resonance imaging enhancement, the patient was diagnosed with a suspected hemorrhagic brain metastasis from lung carcinoma.

**Interventions:**

The patient's family rejected a pathological examination.

**Outcomes:**

The patient's family requested discharge after diagnosis.

**Lessons:**

The present case emphasizes the need to consider hemorrhagic metastasis as a differential diagnosis in patients presenting with solitary intracerebral hemorrhage whose location is uncommon, especially when the poor general state of the patient cannot be attributed to hypertensive intracerebral hemorrhage.

## 1 Introduction

Hemorrhagic brain metastasis (BM) from lung cancer manifests as multiple lesions with large edema and an irregular shape. The presence of both hemorrhagic lesions and non-hemorrhagic lesions in patients with multiple metastases is rare. Here, we report on a patient who presented with a solitary intracerebral hemorrhage, the cause of which was established as lung cancer metastasis. This emphasizes the importance of considering metastasis in a differential diagnosis of patients presented with only intracerebral hemorrhage.

### 1.1 Case Report

**Figure 1:**
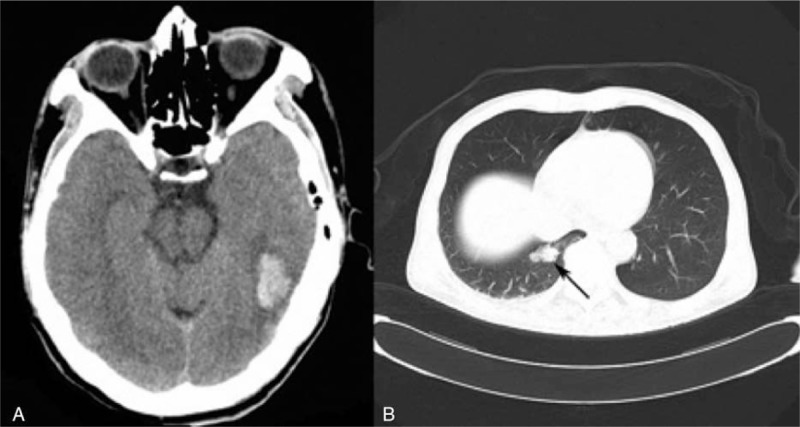
Brain CT and lung CT. Brain CT (A) showed hemorrhage in the left temporal occipital lobe. Lung CT (B) showed a high density lesion in the right posterior lobe. CT = computed tomography.

A 54-year-old man was admitted to the hospital for a severe headache. He reported a sudden headache presented with whole head pain, especially the frontal part, 4 days prior. He showed no signs of blurred vision, diplopia, dizziness, nausea, vomiting, seizures, limb weakness, consciousness disturbance, or a recent, significant change in body weight. He reported no history of hypertension or familial history of cerebral hemorrhage. On admission, his blood pressure was 160/95 mmHg and his neurological examination was normal. The brain computed tomography (CT) scan showed hemorrhage in the left temporal occipital lobe (Fig. 1A). The CT angiography of the intracranial and cervical arteries showed no evidence of vascular malformation, aneurysm, or arteriovenous fistula. Brain magnetic resonance venography (MRV) revealed no obvious abnormalities. Laboratory test results were normal.

After dehydration to reduce intracranial pressure, nerve nutrition, and symptomatic treatment for 1 week did not relieve the symptoms. The general state of the patient, vision, and content of consciousness were significantly decreased. Cerebrospinal fluid (CSF) pressure was >400 mmH_2_O. Protein levels, cell count, and glucose in the CSF were normal and cytology showed no neoplastic cells.

**Figure 2:**
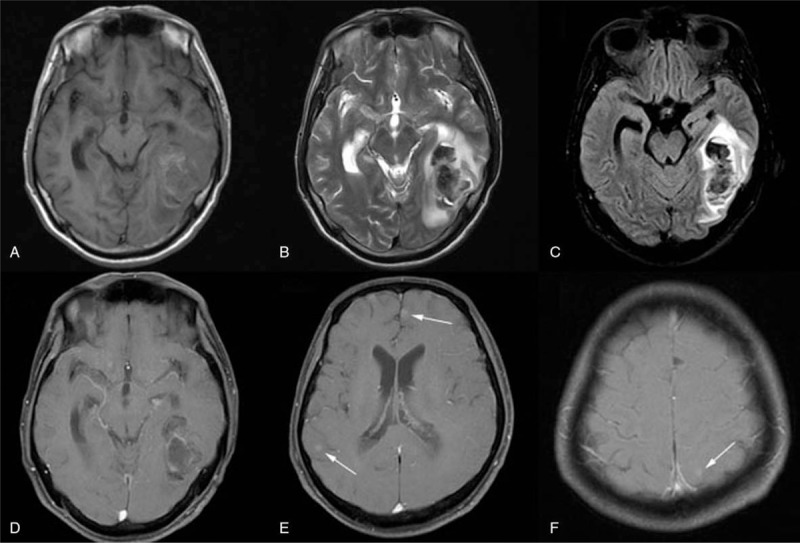
Magnetic resonance imaging enhancement of brain. A–D showed a lesion in the left temporal occipital lobe and bilateral ventricle hemorrhage. E and F, Gadolinium-enhanced T1-weighted image. It shows several enhanced spots on the left frontal, parietal, and right temporal lobes.

Brain magnetic resonance imaging enhancement (Fig. 2) revealed a hemorrhagic lesion in the left temporal occipital lobe and bilateral ventricle, and multiple abnormal bilateral nodules. Lung CT scans revealed a high-density lesion in the right posterior lobe, located at the posterior basal segment of the bronchus, reminiscent of a malignant tumor (Fig. 1B). We administered mannitol, due to high cranial pressure, to reduce intracranial edema, pressure, and pain. We also recommended a pathological examination to define the nature of the lesions; his family refused and requested discharge.

## 2 Discussion

The most common reasons for hemorrhagic BM include melanoma, choriocarcinoma, renal cell carcinoma, and bronchogenic carcinoma.^[1]^ It is rare that hemorrhage occurs in a solitary BM from lung carcinoma. Because of the presence of multiple, abnormal bilateral nodules in both the brain and lungs, we considered hemorrhage to be caused by BM from the lung rather than hypertension. Since the patient refused surgical intervention, the diagnosis could not be confirmed without a biopsy.

The morbidity of BM is higher in patients with lung carcinoma than that of melanoma, breast cancer, renal cell carcinoma, and colorectal cancer.^[2]^ Brain metastasis is found in 10% to 25% of lung cancer patients upon initial diagnosis where 40% to 50% of lung cancer metastasized to the brain during the course of disease.^[3]^ Although an occupied effect and peripheral edema may be seen in intracerebral hemorrhage (ICH), regardless of the cause, the location of bleeding may indicate its underlying etiology.^[4]^

The most common clinical symptom of BM upon initial diagnosis was a headache (25.2%), followed by motor dysfunction (20.5%), dizziness (5.8%), and seizure (3.5%). Cerebral lesions were asymptomatic at the time of the initial diagnosis of BM (50.8%).^[5]^ Since these symptoms are easily confused with ICH, the diagnosis of BM can be missed.

The median survival time for untreated BM patients is 1 to 2 months, which may be extended to 6 months with radiotherapy and chemotherapy.^[6]^ The present case emphasizes the need to consider hemorrhagic metastasis as a differential diagnosis in patients presenting with solitary intracerebral hemorrhage whose location is uncommon, especially when the poor general state of the patient cannot be explained by hypertensive intracerebral hemorrhage. Therefore, early diagnosis and active treatment are vital to improve prognosis and survival.
